# Case report: NUP98::LEDGF fusion gene drives malignant hematological tumor with mixed immunological phenotype

**DOI:** 10.3389/fonc.2024.1396655

**Published:** 2024-09-05

**Authors:** Song Xue, Jia-qi Chen, Tong Wang, Li-na Zhang, Man Chen, Hui-peng Sun, Xing-yu Cao

**Affiliations:** ^1^ Department of Bone Marrow Transplant, Beijing Lu Daopei Hospital, Beijing, China; ^2^ Department of Laboratory Medicine, Hebei Yanda Lu Daopei Hospital, Langfang, China; ^3^ Division of Pathology & Laboratory Medicine, Beijing Lu Daopei Hospital, Beijing, China; ^4^ Department of Bone Marrow Transplant, Hebei Yanda Lu Daopei Hospital, Langfang, China

**Keywords:** NUP98::LEDGF, NUP98 rearrangement, AML, T-LBL, Allo-HSCT, mixed lineage leukaemia gene

## Abstract

This is the first report of NUP98::LEDGF positive malignant hematological tumor expressing T cell and myeloid lineage antigens. Patients carrying this fusion gene have a high relapse rate and a poor prognosis, allo-HSCT may be an option to cure this disease. This patient underwent allo-HSCT, a relapse occurred three months post-transplantation. Subsequent screening at our hospital confirmed the presence of the NUP98::LEDGF fusion gene, salvage therapy was administered, followed by a successful second allo-HSCT. Furthermore, we included eight previously reported cases from the literature for analysis and discuss.

## 1 Introduction

Recurrent rearrangements of the NUP98 gene on chromosome 11p15 have been identified in various hematologic neoplasms, including acute myeloid leukemia (AML), myelodysplastic syndromes (MDS), and T-cell acute lymphoblastic leukemia (T-ALL) (1). To date, a total of 72 documented fusion partner genes of NUP98 in the literature, with ongoing research is continually uncovering new partners (2, 3).The most commonly identified partner gene is NSD1, but some partners are exceedingly rare (4). The LEDGF (Lens Epithelium-derived Growth Factor) gene, also known as PSIP1 (PC4 and SFRS1 interacting protein 1), is situated on 9p22. This gene is responsible for encoding two transcriptional coactivators, p75 and p52, produced via alternative splicing. Notably, LEDGF/p75 is critical in the development of MLL-rearranged leukemia (5).The NUP98::LEDGF fusion gene is exceptionally rare, with only eight case reports identified through an extensive search in the PubMed database (2, 6–12).Previous literature has predominantly reported that this fusion gene-positive hematologic neoplasm manifests as myeloid phenotype, frequently associated with a poor prognosis (2, 7–12).In this article, we present a case report of a patient diagnosed with a malignant hematological tumor, characterized by a mixed immunological phenotype driven by the NUP98::LEDGF fusion gene. The patient underwent unrelated allogeneic hematopoietic stem cell transplantation (allo-HSCT) while in complete remission (CR). However, a relapse occurred three months post-transplantation. Subsequent screening at our hospital confirmed the presence of the NUP98::LEDGF fusion gene. In response, salvage therapy was administered, followed by a second allo-HSCT.

## 2 Case presentation

In March 2022, a 31-year-old male patient presented with symptoms of a low-grade fever and bilateral neck lymphadenopathy and sought medical attention at a local hospital. Comprehensive blood cells analysis revealed a white blood cell count of 4.55×10^9/L, a hemoglobin level of 151 g/L, and a platelet count of 295×10^9/L. However, examination of a peripheral blood smear indicated the presence of 2% immature blood cells. Bone marrow smear analysis revealed that 24% of the cells were myeloblasts. Flow cytometry (FCM) of the bone marrow identified 7.17% of abnormal myeloid progenitor cells expressing CD117, CD34, CD7dim, and HLA-DR. These cells were found to be negative for common fusion genes. Chromosomal analysis revealed a karyotype of 46, XY [20]. A bone marrow biopsy confirmed features of myeloid leukemia. Pathological examination of the lymph node biopsy showed diffuse proliferation of morphologically similar small lymphocytes, resulting in the loss of normal lymph node structure. Immunohistochemistry indicated the absence of CD10 and CyclinD1, and the presence of CD3, CD5, Ki67 (index: 80%) and TdT. PET-CT imaging revealed enlarged lymph nodes in multiple anatomical regions, including the bilateral carotid sheath, bilateral posterior cervical spaces, bilateral submandibular regions, submental region, bilateral supraclavicular regions, mediastinum, bilateral axillary regions, along the abdominal aorta, bilateral iliac artery pathways, and bilateral inguinal regions. These lymph nodes also demonstrated increased FDG uptake. Based on comprehensive diagnostic investigations, the patient was diagnosed with combined acute myeloid leukemia and T-cell lymphoblastic lymphoma. Following the initial diagnosis, CHOP chemotherapy in combination with the Ara-c regimen was administered, leading to bone marrow remission after the initial chemotherapy, the patient underwent consolidation therapy with the Hyper CVAD-B regimen. The patient’s bone marrow remains in remission, and subsequent PET-CT imaging indicates complete metabolic remission.

The patient underwent allo-HSCT from an unrelated donor with a perfect 10/10 HLA match. This procedure was performed on July 4, 2022, at a local transplant center. The conditioning regimen include a modified busulfan-cyclophosphamide protocol with anti-human thymocyte immunoglobulin (ATG), comprising: Ara-c (4g/m^2^/d) intravenously from days -10 to -9; busulfan(3.2 mg/kg/d) intravenously from days -8 to -6; Cyclophosphamide (1.8g/m^2^/d) intravenously from days -5 to -4; Me-CCNU (250 mg/kg/d) orally on day -3; and ATG (2.5 mg/kg/d, Genzyme) administered over 4 days from days -5 to -2. For prophylaxis against acute graft-versus-host disease, the patient was administered cyclosporin A, mycophenolatemofetil, and short-term methotrexate. Successful engraftment of white blood cells and platelets was achieved in the patient. At +1 month and +2 months post-transplant, the patient’s bone marrow exhibited morphological complete remission and negative results in flow cytometry.

**Figure 1:**
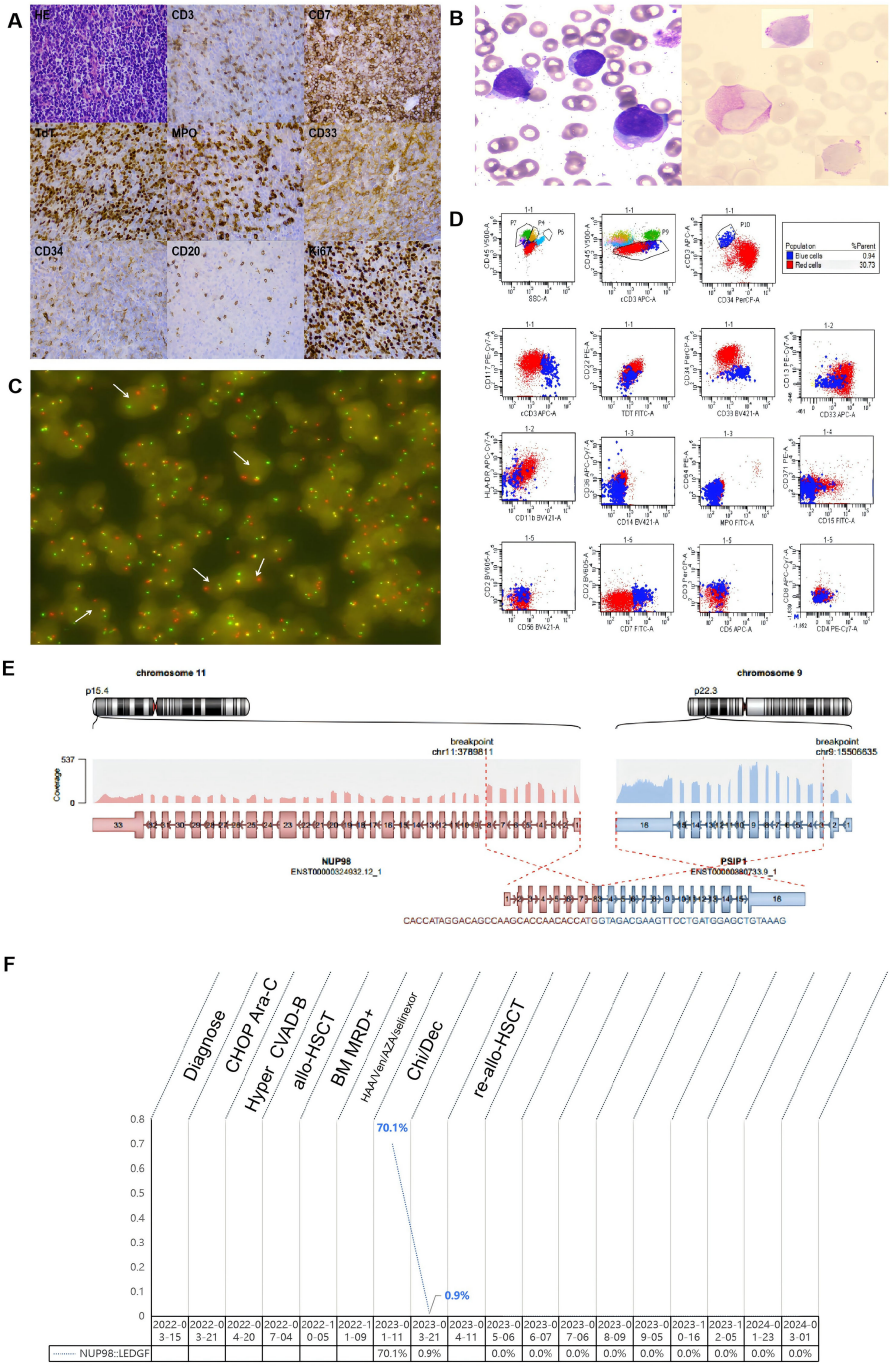
Pathology,laboratory examination results and entire treatment course of the patient. **(A)** HE staining revealed pathological alterations in the patient’s lymph nodes, characterized by a loss of the typical lymph node architecture and a diffuse infiltration of medium-sized, atypical lymphocytes.These atypical lymphocytes featured scanty cytoplasm, round nuclei containing fine chromatin, and inconspicuous nucleoli.Immunohistochemical analysis revealed expression of CD7, TdT, MPO, CD33, and Ki67 in the atypical lymphocytes, along with weak CD3 and CD34 expression, and a lack of CD20 expression. **(B)** In bone marrow smears, the presence of abnormal blast cells is discernible, and PAS staining demonstrates the deposition of glycogen within these cells. **(C)** FISH analysis with the NUP98 separation probe distinctly reveals the presence of the NUP98 separation signal, indicated by a white arrow. **(D)** Flow cytometry analysis of bone marrow identified two distinct subpopulations of leukemia cells:(1) A major clonal tumor subgroup, labeled as the ‘red group’, comprising 30.73% of nucleated cells,expressing CD34, CD38dim, TDT, CD117, CD33bright, CD13, and partially expressing HLA-DRdim, CD7 and cCD3dim, not expressing MPO, CD36, CD14, CD42a, CD15, CD371, CD11b, CD2, CD5, CD3, CD56, CD4 and CD8. This confirmed the classification of the ‘red group’ as a malignant primitive cell subset, displaying both myeloid and T-lineage markers. (2)A second distinct population of small clonal tumor cells, designated the ‘blue group’, accounted for 0.94% of nucleated cells. These cells expressing CD7, cCD3, TDT and CD38, with partial expression of CD117 and CD33dim, not expressing CD34, HLA-DR, CD13, MPO, CD36, CD14, CD64,CD42a, CD15, CD371, CD11b, CD2, CD3,CD5, CD56, CD4, and CD8. This confirmed the ‘blue group’ as malignant precursor T-cell exhibiting myeloid markers. **(E)** RNA sequencing revealed an in-frame fusion event between exon 8 of NUP98 and exon 3 of LEDGF. **(F)** The entire treatment course of the patiet. CHOP, cyclophosphamide, Adriamycin, vincristine, prednisone; Hyper CVAD-B, methotrexate, cytarabine; allo-HSCT, allogeneic hematopoietic stem cell transplantation; BM, bone marrow; MRD, Minimal residual disease; HAA, Homoharringtonine, aclarubicin, cytarabine; Ven, Venetoclax; AZA, Azacytidine; Chi, chidamide; Dec, decitabine.

On October 5, 2022 (+3 months post-transplant), FCM identified 0.51% abnormal myeloid blast cells, prompting the discontinuation of cyclosporine administration. Subsequently, on October 21, 2022, a reexamination of bone marrow via FCM revealed an increase to 0.66% abnormal myeloid blast cells. The patient commenced a chemotherapy regimen comprising HAA (homoharringtonine, aclacinomycin, and cytarabine), venetoclax, and azacitidine on November 9, 2022. During treatment, the patient experienced multiple episodes of infectious fever, necessitating the cessation of chemotherapy. Subsequently, the patient was treated with oral venetoclax and selinexor; however, this treatment was ineffective, and as evidenced by a continued increase in the proportion of abnormal bone marrow cells. The patient was then referred to our hospital for advanced treatment, where a comprehensive evaluation of their condition was conducted. To establish a definitive diagnosis, a pathological reevaluation of the initial lymph node biopsy specimen was performed. This reevaluation confirmed the presence of a malignant lymphohematopoietic tumor expressing T cell and myeloid lineage antigens (**Figure 1A**).

On February 11, 2023, the patient underwent bone marrow aspiration at our hospital. Examination of bone marrow smears (**Figure 1B**) revealed the presence of abnormal blast cells, which comprised 31.5% of the total cellular population. FCM analysis of the bone marrow sample (**Figure 1C**) revealed 31.67% of malignant, immature cells that co-expressed markers of both myeloid and T-cell lineages. Genetic mutation analysis of the bone marrow sample identified specific mutations, namely PHF6 R274* (33%), JAK3 V674A (16%), CXCR4 V320Efs23 (15%), CREBBP G1418S (14%), NOTCH1 L2311Tfs29 (10%), and KMT2D P1669A (6%). RNA sequencing analysis (**Figure 1D**) confirmed the presence of the NUP98-LEDGF fusion gene. Fluorescence *in situ* hybridization (FISH) was conducted on the initial lymph node biopsy specimen. The FISH analysis revealed a disruption in the NUP98 gene, as depicted in **Figure 1E**.

The patient was prescribed chidamide and decitabine as part of a chemotherapy regimen. On March 21, 2023, bone marrow examination confirmed the patient was in a state of remission. FCM analysis revealed no malignant immature cells. Given the patient’s premature relapse following the initial allo-HSCT and the unfavorable prognosis associated with this specific malignant hematological tumor, a decision was made by the patient to proceed with a second allo-HSCT. From April 9-11, 2023, the patient underwent allo-HSCT using an 8/10 HLA-matched sibling donor. The conditioning regimen included total body irradiation (TBI) and ATG, comprising: TBI (200 cGy bid) from days -11 to -9; Ara-c (3g/m^2^/d) intravenously from days -8 to -6; fludarabine (30mg/m^2^/d) intravenously from days -5 to -2; and ATG-Pig (20 mg/kg/d, Sinopharm) from days -5 to -2. Prophylaxis for GVHD was administered using tacrolimus, mycophenolatemofetil, and short-term methotrexate. The patient achieved neutrophil engraftment on day +17 and platelet engraftment on day +12. Post-transplantation assessments consistently showed negative results for NUP98::LEDGF gene quantification. As of the latest follow-up, the patient remains disease-free (**Figure 1F**). Following second allo-HSCT, patients consistently reported an improved quality of life, with a gradual resumption of routine social activities. The patient provided written informed consent for publication of this report and accompanying images.

## 3 Literature review and discussion

The NUP98::LEDGF fusion gene was initially identified in 1994 in a case involving a 20-year-old female patient with biphenotypic leukemia. Since then, there have been a total of eight cases have been reported in the literature spanning from 1994 to 2019 (**Table 1**). The patient population demonstrates an almost equal male-to-female ratio of 4:5. At diagnosis, the median age among patients was 52 years (range 5‐64), and the median WBC count stood at 8.63 (range 1.0‐293) × 10^9^/L. The majority of initial disease diagnoses comprised myeloid neoplasms, including five cases of acute myeloid leukemia, one case of myelodysplastic syndrome, and one case of chronic myelogenous leukemia blast crisis. The remaining cases exhibited B-lineage and myeloid involvement, while the present case exhibits a complex immunological phenotype involving both T lineage and myeloid involvement. In the reported eight cases, the typical karyotypic abnormality 46,XX, t(9;11) (p22; p15), was observed. However, the current case did not exhibit typical karyotypic abnormalities but rather presented a normal karyotype, possibly due to NUP98 rearrangements often involving cryptic rearrangements that are not detected by chromosomal karyotyping analysis (1, 13). Additionally, the first reported case also presented with a normal karyotype at relapse (6). Some NUP98 translocations have been linked to secondary leukemias resulting from topoisomerase inhibitors (12),yet all nine reported cases of NUP98::LEDGF malignant hematological tumors reported thus far have been *de novo*.

**Table 1:** Summary of NUP98::LEDGF+ patients.

Author	Time	Sex	Age	WBC (10^9^/L)	Leukemia type	Karyotype	NUP98-LEDGF fusion	Molecular biology abnormalities	Treatment	HSCT	Survival status	Overall survival
Ha SY (6)	1994	female	20	63.8	Biphenotypic leukemia(B/M)	46,XX, t(9;11) (p22; p15)	NA	NA	Chemotherapy	NO	NO	3months
Ahuja HG (9)	2000	male	52	50.5	AML-M1	46,XX, t(9;11) (p22; p15)	N9/L7	NA	Chemotherapy	YES	NO	9months
Hussey DJ (10)	2001	female	60	1.5	AML- M2	46,XX,t(9;11)(p22;p15) [13]/46,XX [2]	N8/L2	NA	Chemotherapy	NO	NO	54months
Morerio C (8)	2005	female	5	207	AML-M3v	46,XX, t(9;11) (p22; p15)	N9/L5	NA	Chemotherapy All trans retinoic acid	NO	NO	NA
Grand FH (11)	2005	male	29	293	CML-BC(AML)	46,XY, t(9;22)(q34;q11.2) [3]/ 46,XY, t(9;22),t(9;11)(p21;p15) [16]/46,XY, t(9;22),t(9;11)(p21;p15),idem, -18 [1]	N9/L7	NA	Chemotherapy Imatinib	NO	NO	1.5months
Lundin C (12)	2010	female	64	2.5	AML-M2	46,XX,t(9;11)(p22;p15)[24]/46,XX[1]	NA	FLT3-ITD	Chemotherapy	NO	NO	24months
Yamamoto K (7)	2012	female	64	1.8	MDS-EB-2	46,XX,t(9;11)(p22;p15)[20]	N11/L8 N12/L8	FLT3-ITD	NA	YES	NO	7.5months
Gallego Hernanz MP (2)	2019	male	58	1.0	AML-M2	46,XY,t(9;11)(p22;p15)[35]/46,XY[2]	N9/L10	IDH1 SRSF2 WT1	Chemotherapy	YES	YES	>31months
Present case	2023	male	31	8.63	Biphenotypic leukemia(T/M)	46,XY[20]	N8/L3	PHF6 JAK3 CXCR4 CREBBP NOTCH1 KMT2D	Chemotherapy	YES	YES	>22months

WBC, white blood cell count; HSCT, hematopoietic stem cell transplantation; NA, not available; AML, acute myeloid leukemia.

The recruitment of NUP98 fusion proteins to their target genes is mediated by the mixed lineage leukemia (MLL) complex, via a direct interaction between MLL and Menin (14). LEDGF/p75 is not essential for steady-state hematopoiesis, but it plays a critical role in the initiation of MLL-mediated leukemia (5).The NUP98-LEDGF transcript encodes a protein that merges the amino terminus of the NUP98 gene, comprising 28 out of 38 FG repeats, with LEDGF p52/75. FG repeats in NUP98 fusion proteins are known to serve as transactivation domains, capable of recruiting CREBBP/EP300 (1). This indicates the possibility that the fusion proteins could function as atypical transcription factors. Our present study, in conjunction with previously published data, demonstrates notable variation in breakpoint locations within both NUP98 and LEDGF, a hallmark of NUP98-LEDGF fusions (2, 7–11). For NUP98, the primary breakpoint is located in exon 9, followed by exon 8. Conversely, the breakpoints for LEDGF are widely dispersed, spanning multiple exons of LEDGF (**Table 1**). The breakage of LEDGF results in the loss of its nucleolus localization signal, similar to NPM mutations. Consequently, NUP98‐LEDGF fusions display significant variability among patients. This could potentially account for the variations in immune phenotypes and clinical outcomes seen in different patients, including the patient we reported.

Despite their heterogeneous clinical and biological features, NUP98-LEDGF hematological malignancies often manifest as highly aggressive disorders and are associated with a poor prognosis. While the majority of patients have undergone proactive treatment, including hematopoietic stem cell transplantation, only two cases of long-term disease-free survival have been reported in this group, with a median survival time of 15.5 (range 1.5‐54) months. Genetic mutation results were available for four patients, two of whom exhibited FLT3-ITD mutation. This similarity might also be present in other leukemias with NUP98 rearrangements, providing a potential target for targeted therapy (15).Generally, in case of NUP98-LEDGF fusion, allo-HSCT should regarded as a viable treatment option, contingent upon the patient’s age, genetic factors, and response to initial conventional chemotherapy. The patient in our report underwent allo-HSCT in CR, but experienced early disease progression post-transplantation. This case is not unique among the few reported patients with NUP98-LEDGF (7), further underscoring a relatively poor prognosis for NUP98-LEDGF-related hematological malignancies. The patient is presently in disease-free survival, having demonstrated NUP98-LEDGF negativity after a second allo-HSCT. Monitoring the transcription levels of NUP98-LEDGF is crucial for assessing minimal residual disease in NUP98-LEDGF-related hematological malignancies, which are associated with a poor prognosis.

## Data Availability

The original contributions presented in the study are included in the article/supplementary material, further inquiries can be directed to the corresponding author/s.
